# Various response of *Pinus tabulaeformis* Carr. regeneration in artifical gaps

**DOI:** 10.1038/s41598-017-15322-8

**Published:** 2017-11-06

**Authors:** Zhibin Wang, Kuangji Zhao, Haijiao Yang, Lvyi Ma, Zhongkui Jia

**Affiliations:** 1 Key Laboratory for Silviculture and Conservation of Ministry of Education, College of Forestry, Beijing Forestry University, No. 35, Qinghua East Road, Beijing, 100083 P. R. China; 2 National Energy R & D Center for Non-Food Biomass, Beijing Forestry University, No. 35, Qinghua East Road, Beijing, 100083 P. R. China; 3 National Engineering Laboratory for Tree Breeding, College of Biological Sciences and Technology, Beijing Forestry University, No. 35, Qinghua East Road, Beijing, 100083 P. R. China

## Abstract

Understanding the influence of gaps in promoting canopy recruitment will help to maintain structural stability and achieve continuous forest cover. We established three control plots and experimental plots with three replications each (gap sizes L-I, L-II, L-III, and L-IV) in a Chinese pine (*Pinus tabulaeformis* Carr.) plantation to test the short-term effects of gap size on the age distribution, density and growth, and annual height and ground diameter growth for regeneration established before (REBG) and after (REAG) gap creation. Age distribution exhibited an approximately normal distribution, with the numbers of REBG and REAG decreasing and increasing, respectively, as the age increased. Although there was no difference in density among gap size classes, regeneration growth positively responded to gap size, with maximum values observed in class L-III. Annual average height growth after (AAH-A) gap creation was significantly greater than that before (AAH-B) gap creation for REBG among gap sizes, suggesting that gaps promote the rapid growth of regeneration. However, the responses of height and ground diameter growth in REBG to gap size were not immediate and exhibited a response delay of 2–4 years. Similarly, for the height and ground diameter growth of REAG, significant differences were first observed within years 2–4 after germination in the same growing season for all gap size classes.

## Introduction

In recent decades, forest management practices have resulted in the transformation of even-aged forests, especially coniferous forests, into uneven-aged or mixed forests^1,2^. The use of gaps to successfully promote structural transformations is considered a primary silvicultural treatment that can be used to maintain dynamic stability and sustainable timber production^3,4^. Opening gaps in the forest canopy not only creates opportunities for seed germination and seedling establishment, survival and growth^4^ but also influences species composition and size structure by altering the characteristics of micro-habitats, including the availability of light^2,5^. For example, saplings growing under a canopy reach a critical size with a negative carbon balance, which may cause mortality as their sizes continue to increase while light declines^6^ but can be relieved through gap creation^7^. Some studies suggest that the low-light produced in small harvested gaps contribute to the establishment and growth of shade-tolerant species^2^, whereas large harvested gaps favor moderately shade-tolerant species^8,9^. Vilhar *et al*.^10^ reported that opening circular gaps with diameters larger than the stand height increases the coverage of ground vegetation and hinders the regeneration of moderately shade-tolerant species. Briefly, whether the existence of an optimum gap size to further establishment, growth and persistence of regeneration or whether gaps could successfully promote canopy recruitment to achieve continuous forest cover in a given species should be taken into account when promoting gap creation.

Chinese pine (*Pinus tabulaeformis* Carr.) is an endemic species with strong adaptability in China. It can mix with some broadleaf trees to form a good stand structure and be treated as the main timber species, and it plays an important role in soil and water conservation and vegetation restoration in northern China^11,12^. However, most Chinese pine plantations planted by the government in the 1960s–1970s suffer from slow growth, pest and disease outbreaks, and declining soil fertility resulting from the simplicity and maturity of the stands therein. Therefore, the main purpose of this study is to test the effects of canopy gaps on Chinese pine regeneration to provide a scientific reference for the heterogeneity and multi-layer structure of plantations. Previous studies have investigated Chinese pine regeneration in gaps. For instance, Wang *et al*.^13^ found that the heights of seedlings and saplings significantly increased when gaps with diameters 0.75 to 1.25 times the mean stand height were created. Wang and Liu^2^ illustrated that opening gaps not only increased the regeneration density but also led to some regeneration becoming dominant in specific gap sizes. These findings were similar to those of Dong *et al*.^14^, who tested the responses of natural regeneration to gap sizes (4–40 m^2^) in a stand with a canopy density of 0.7 and found that density, height and ground diameter of regeneration in gaps were 2.5–7.0, 1.0–1.7 and 1.0–1.6 times greater than that which occurred under the canopy, respectively. Other studies have come to similar conclusions^15,16^. However, little is known about how the age of regeneration is distributed in different gap sizes and whether an optimum gap size to promote Chinese pine regeneration exists. Additionally, whether the growth of regeneration established before gap creation responds immediately to the effects of gaps or exhibits a response delay remains unknown.

To better understand the extent to which gap size affects regeneration in a Chinese pine plantation, the Chinese pine regeneration in gaps was divided into two categories: REBG refers to Chinese pine regeneration established before gap creation, and REAG refers to Chinese pine regeneration established after gap creation. The objectives of this research were to: (i) assess the age distribution of regeneration in different gap sizes, (ii) identify changes in density and growth of REAG and REBG in response to different gap sizes, and (iii) determine whether the height and ground diameter growth of REAG and REBG respond immediately to the creation of gaps.

## Results

### Age distribution

**Figure 1:**
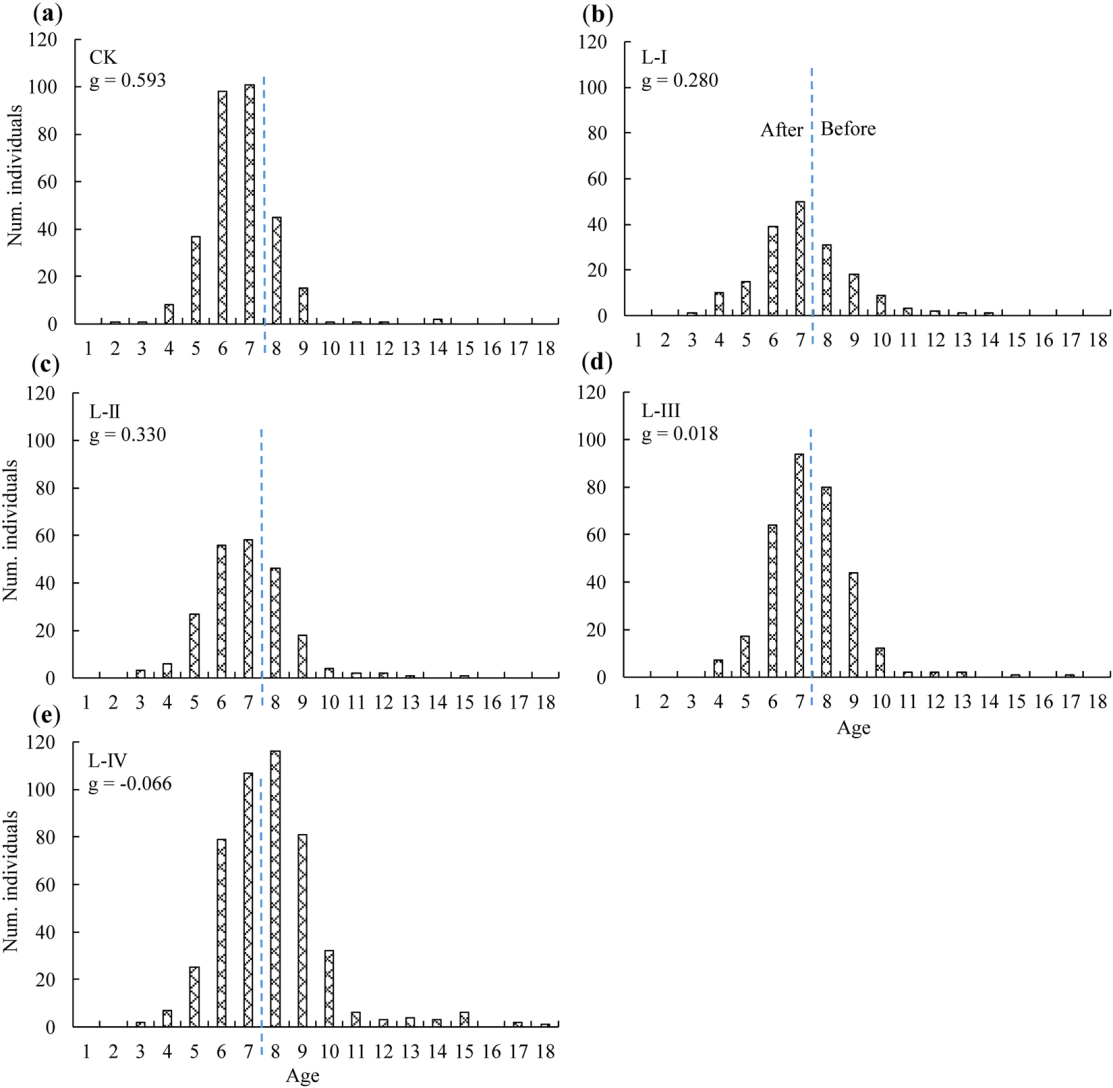
Age distribution of Chinese pine regeneration in different gap size classes. The coefficient of skewness (*g*) of each gap size class is shown. The vertical dashed lines represent the time of gap creation. The regeneration on the left and right sides of the line was established after (REAG) and before (REBG) gap creation, respectively. L-I (5–20 m^2^), L-II (20–40 m^2^), L-III (40–60 m^2^), and L-IV (60–100 m^2^) are the different gap size classes. CK = control plots.

A frequency histogram of regeneration age for the different gap size classes exhibited an approximately normal distribution, with the peak at the age of seven (Fig. 1a–d) for all classes except class L-IV, where the peak occurred at the age of eight after the creation of gaps (Fig. 1e). The age of regeneration was mainly concentrated between 4 ≤ age ≤ 10, with most individual trees between six and eight years old. However, regeneration in the class L-IV were between seven and nine years old. In addition, the amounts of REAG and REBG decreased and increased, respectively, as age decreased for all gap size classes (Fig. 1). To examine the differences in the age distribution among gap size classes, we conducted a Kolmogorov-Smirnov test. The results revealed prominent differences in the age distributions between CK and different gap sizes. Significant differences were also found between all pairs of gap size classes excluding the differences between L-I and L-II, and between L-I and L-III (Table 1). L-IV was the only gap size class with a coefficient of skewness, *g* < 0 (−0.066), which indicated the presence of fewer large regenerated trees than small ones. Moreover, the tail toward older individuals increased as the gap size increased, and vacant age-distribution classes were noted, such as the age of 13 for CK, 14 for L-II, 14 for L-III, and 16 for L-IV, respectively (Fig. 1).

**Table 1:** The Kolmogorov-Smirnov tests for the age distributions in different gap size classes.

	CK	L-I	L-II	L-III	L-IV
Age (year)					
CK	---				
L-I	Z = 1.624	---			
	***p*** ** < 0.001**				
L-II	Z = 1.385	Z = 0.638	---		
	***p*** **= 0.008**	*p* = 0.382			
L-III	Z = 2.936	Z = 0.982	Z = 1.622	---	
	***p*** ** < 0.001**	*p* **= **0.084	***p*** ** < 0.001**		
L-IV	Z = 4.479	Z = 1.996	Z = 2.535	Z = 1.318	---
	***p*** ** < 0.001**	***p*** ** < 0.001**	***p*** ** < 0.001**	***p*** ** = 0.009**	

Significant results (*p* < 0.01) are highlighted in bold. Abbreviations are the same as those provided in Fig. 1.

**Figure 2:**
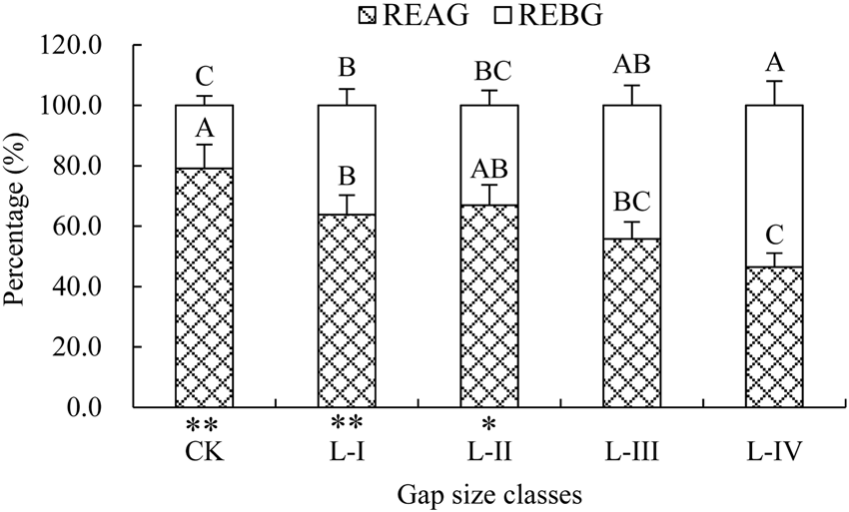
The percentages of REAG and REBG in the same gap size class. REAG = regeneration that was established after gap creation; REBG = regeneration that established before gap creation. Abbreviations are the same as those provided in Fig. 1. The bars represent ± SE. Uppercase (*p* < 0.01) letters above the standard error bars indicate significant differences (Duncan’s test) in the percentages of REAG and REBG among gap size classes. Asterisks (**p < 0.01; *p < 0.05) under the *X*-axis indicate significant differences (Duncan’s test) between the percentages of REAG and REBG in the same gap size class.

The age distribution of regeneration varied with gap size class. Moreover, the percentages of REBG and REAG among gap size classes were analyzed further, and the maximum REAG was observed in CK (*p* < 0.01, 79.10%). A downward trend was observed as gap size increased. In contrast, the most significant percentage of REBG was found in L-IV (*p* < 0.01, 53.59%) and gradually increased along the gap size gradient, although the percentages of REBG in CK (*p* < 0.01), L-I (*p* < 0.01) and L-II (*p* < 0.05) were significantly smaller than those of REAG in the same gap size class (Fig. 2).

### Density and growth

The densities of ALL and REAG declined as gap size increased, although the differences were not significant. On the other hand, the maximum and minimum REBG densities occurred in L-IV (0.57 trees m^−2^) and CK (0.22 trees m^−2^), respectively (Table 2). For ALL, REAG and REBG, the growth parameters, including height, ground diameter, crown, CHG, AAH, and AAGD, reached their maximum values in class L-III, increasing from CK to L-III and then decreasing in L-IV. All the maximum growth values occurred in L-III and were 1.5–2.0 times the minimum value, which was found in CK. Excluding the AAGD, in L-IV, the aforementioned parameters did not differ substantially from those identified in L-III. In addition, no differences were observed between the growth parameters of L-I and CK, irrespective of the CHG and AAGD of ALL and the height and CHG of REBG (Table 2).

**Table 2:** Density and growth parameters statistics for ALL, REAG, and REBG in response to gap size classes.

	CK	L-I	L-II	L-III	L-IV	*p*-value
**Density (tree m** ^**−2**^ **)**						
All	1.04 ± 0.03	1.36 ± 0.23	1.18 ± 0.11	1.07 ± 0.10	1.06 ± 0.27	0.667
REAG	0.82 ± 0.04	0.87 ± 0.18	0.79 ± 0.09	0.60 ± 0.15	0.49 ± 0.17	0.192
REBG	0.22 ± 0.04	0.49 ± 0.06	0.39 ± 0.07	0.47 ± 0.09	0.57 ± 0.11	0.064
**Height (cm)**						
All	80.86 ± 4.16^C^	97.66 ± 2.01^BC^	101.23 ± 4.61^B^	123.27 ± 4.37^A^	113.27 ± 6.32^AB^	**0.003**
REAG	62.93 ± 2.32^b^	70.53 ± 3.20^b^	76.97 ± 3.48^b^	93.93 ± 7.89^a^	77.84 ± 4.97^b^	**0.012**
REBG	98.79 ± 7.73^C^	124.80 ± 1.58^B^	125.49 ± 7.93^B^	152.62 ± 9.41^A^	148.70 ± 10.43^AB^	**0.005**
**Ground diameter (cm)**						
All	1.36 ± 0.06^c^	1.60 ± 0.09^bc^	1.83 ± 0.15^ab^	2.22 ± 1.00^a^	2.03 ± 0.09^ab^	**0.018**
REAG	1.03 ± 0.04	1.16 ± 0.07	1.40 ± 0.06	1.67 ± 0.24	1.33 ± 0.14	0.051
REBG	1.70 ± 0.10^b^	2.03 ± 0.13^b^	2.26 ± 0.25^ab^	2.78 ± 0.31^a^	2.73 ± 0.16^a^	**0.018**
**Crown (cm)**						
All	44.56 ± 2.72^C^	50.35 ± 1.33^BC^	58.37 ± 2.66^AB^	69.20 ± 2.61^A^	61.27 ± 3.53^AB^	**0.003**
REAG	31.09 ± 1.54^D^	34.40 ± 0.49^CD^	43.93 ± 2.59^AB^	51.60 ± 3.97^A^	40.03 ± 2.35^BC^	**0.001**
REBG	58.03 ± 4.39^c^	66.31 ± 2.43^bc^	72.81 ± 3.59^bc^	86.79 ± 6.54^a^	82.51 ± 6.78^ab^	**0.014**
**Current height growth (CHG, cm)**						
All	16.85 ± 0.78^C^	23.87 ± 2.11^B^	25.39 ± 1.01^B^	32.77 ± 1.56 ^A^	28.72 ± 1.21 ^AB^	**0.001**
REAG	15.91 ± 1.10^b^	19.36 ± 1.41^b^	22.01 ± 1.39^ab^	28.01 ± 3.29^a^	21.59 ± 2.25^ab^	**0.022**
REBG	20.34 ± 1.13^C^	28.84 ± 1.81^B^	31.79 ± 1.55^B^	38.70 ± 2.83^A^	34.22 ± 0.87^AB^	**0.000**
**Annual average height growth (AAH, cm)**						
All	10.74 ± 0.44^C^	12.44 ± 0.24^BC^	13.46 ± 0.56^B^	16.01 ± 0.52^A^	14.01 ± 0.66^AB^	**0.003**
REAG	10.08 ± 0.38^C^	11.16 ± 0.31^BC^	12.47 ± 0.49^AB^	14.57 ± 1.11^A^	12.09 ± 0.71^BC^	**0.009**
REBG	11.39 ± 0.63^C^	13.72 ± 0.35^BC^	14.45 ± 0.88^B^	17.44 ± 1.06^A^	15.92 ± 0.93^AB^	**0.004**
**Annual average ground diameter growth (AAGD, cm)**						
All	0.17 ± 0.00^D^	0.20 ± 0.01^C^	0.24 ± 0.01^B^	0.27 ± 0.01^A^	0.25 ± 0.01^B^	**0.000**
REAG	0.17 ± 0.00^C^	0.18 ± 0.01^C^	0.23 ± 0.01^B^	0.25 ± 0.01^A^	0.22 ± 0.01^B^	**0.000**
REBG	0.20 ± 0.01^C^	0.22 ± 0.01^C^	0.26 ± 0.01^B^	0.30 ± 0.01^A^	0.28 ± 0.01^B^	**0.000**
**The ratio of height and ground diameter (H/GD, cm cm** ^**-1**^ **)**						
All	63.58 ± 1.04^AB^	65.98 ± 1.52^A^	58.27 ± ± 1.08^C^	62.01 ± 1.19^B^	60.11 ± 1.06^BC^	**0.001**
REAG	64.38 ± 1.18^AB^	67.19 ± 2.59^A^	57.85 ± 1.40^C^	64.22 ± 1.88^AB^	60.68 ± 1.52^BC^	**0.003**
REBG	60.59 ± 2.15	64.86 ± 1.67	59.12 ± 1.62	59.23 ± 1.25	59.62 ± 1.47	0.175

REAG = regeneration that established after gap creation; REBG = regeneration that established before gap creation; ALL = all regeneration in a gap, representing the sum of REAG and REBG. Abbreviations are the same as those provided in Fig. 1. Values are presented as the mean ± SE. Means followed by different uppercase (*p* < 0.01) or lowercase (*p* < 0.05) letters indicate significant differences (Duncan’s test) among gap size classes. Significant results (*p* < 0.05) are highlighted in bold.

**Figure 3:**
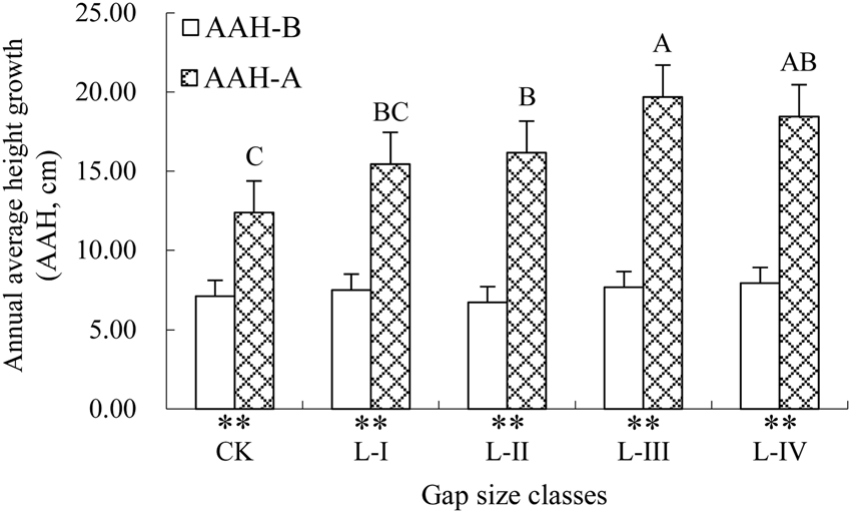
The AAH-B and AAH-A of REBG among gap size classes. The bars represent ± SE. Uppercase (*p *< 0.01) letters above the standard error bars indicate significant differences (Duncan’s test) in AAH-A among gap size classes. Asterisks (**p < 0.01) under the *X*-axis indicate significant differences (Duncan’s test) between AAH-B and AAH-A in the same gap size class. AAH-B = the annual average height growth before gap creation; AAH-A = the annual average height growth after gap creation. Other abbreviations are the same as those provided in Fig. 1.

To compare the AAH-B and AAH-A of REBG (Fig. 3), the growth of regeneration in CK was also divided into two stages (before and after gap creation) according the year of gap creation. The results showed that the AAH-B values were small and did not differ significantly among gap size classes (*p* = 0.054), ranging from 6.72 to 7.94 cm. In contrast, the AAH-A exhibited significant differences among gap size classes (*p* < 0.01), increasing from CK to L-III and then decreasing in L-IV, as noted for the other growth parameters analyzed above. The largest (19.70 cm) and smallest (12.38 cm) growth occurred in L-III and CK, respectively. Additionally, AAH-A was always 1.7–2.6 times larger than AAH-B in the same gap size (Fig. 3), suggesting that gap creation can rapidly promote the growth of regeneration.

### Annual height and ground diameter growth

**Figure 4:**
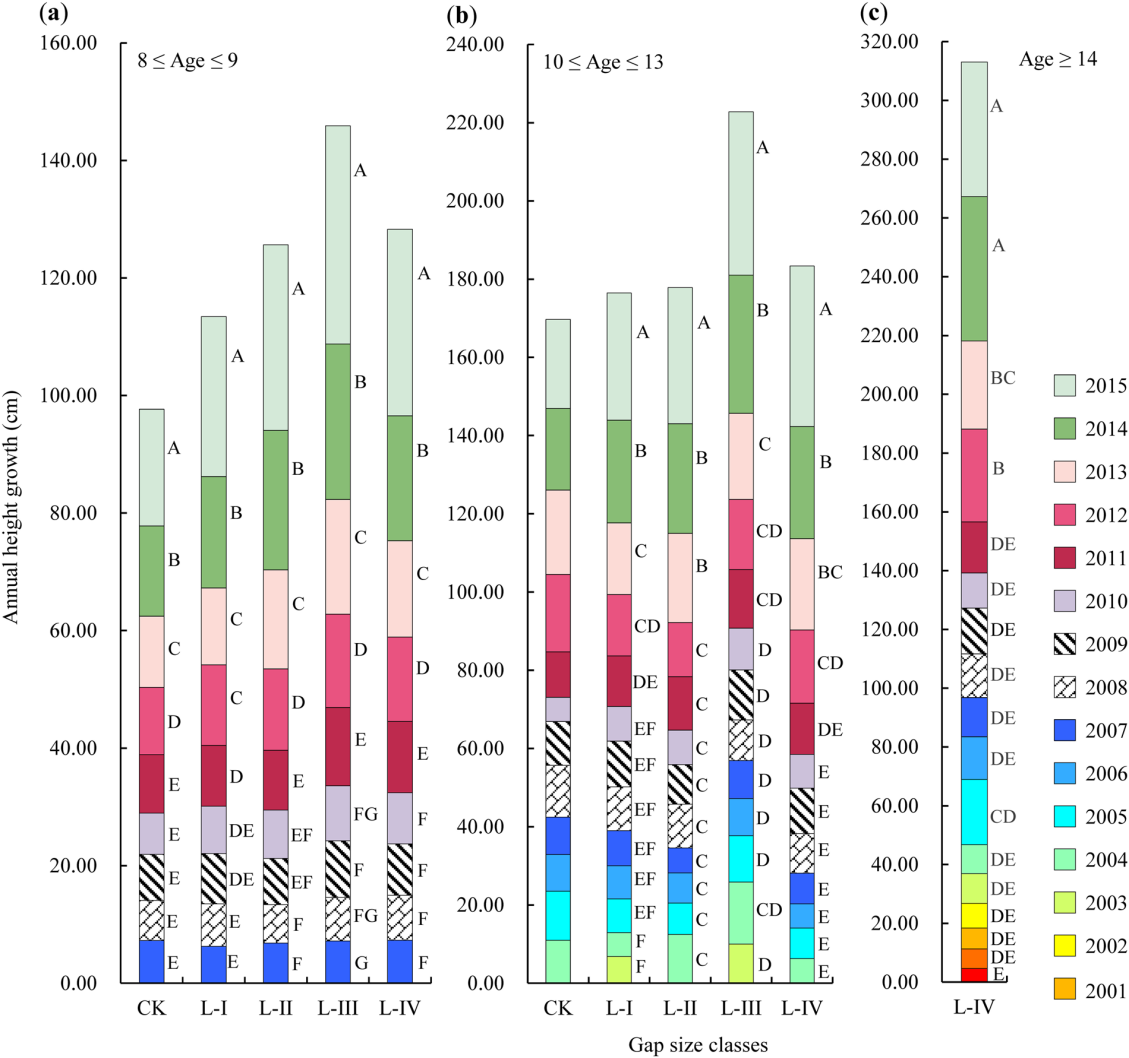
The response of the annual height growth of REBG to gap size. (**a**) REBG of 8 ≤ age ≤ 9. (**b**) REBG of 10 ≤ age ≤ 13. (**c**) REBG of age ≥ 14. The stacked histograms represent the height growth of each growing season for REBG in different gap size classes. The uppercase (*p* < 0.01) letters immediately to the right of each histogram indicate significant differences (Duncan’s test) in height growth in different growing seasons. The tested gaps in this study were created in the winter of 2008. Abbreviations are the same as those provided in Fig. 1.

The response delay in the annual height growth of REBG differed among gap sizes and age classes (Fig. 4). For the regeneration with 8 ≤ age ≤ 9, the first significant height growth occurred in 2011 (i.e., 3 years after gap creation) for regeneration in L-I, L-II, L-III and L-IV, whereas in CK, the first significant height growth occurred in 2012 (i.e., 4 years after gap creation) (Fig. 4a). For regeneration with 10 ≤ age ≤ 12 (or 13), no obvious differences in the annual height growth were observed in CK, but the first significant growth occurred in 2012 in L-I and L-IV. In contrast, a response delay was noted in L-II and L-III, with the first significant height growth recorded in 2013 (i.e., 5 years after gap creation) (Fig. 4b). Finally, regeneration with age ≥ 14 only appeared in L-IV, and the height growth of these regeneration also had a 4-year response delay in response to the gap effect (i.e., the first discrepancy occurred in 2012) (Fig. 4c).

**Figure 5:**
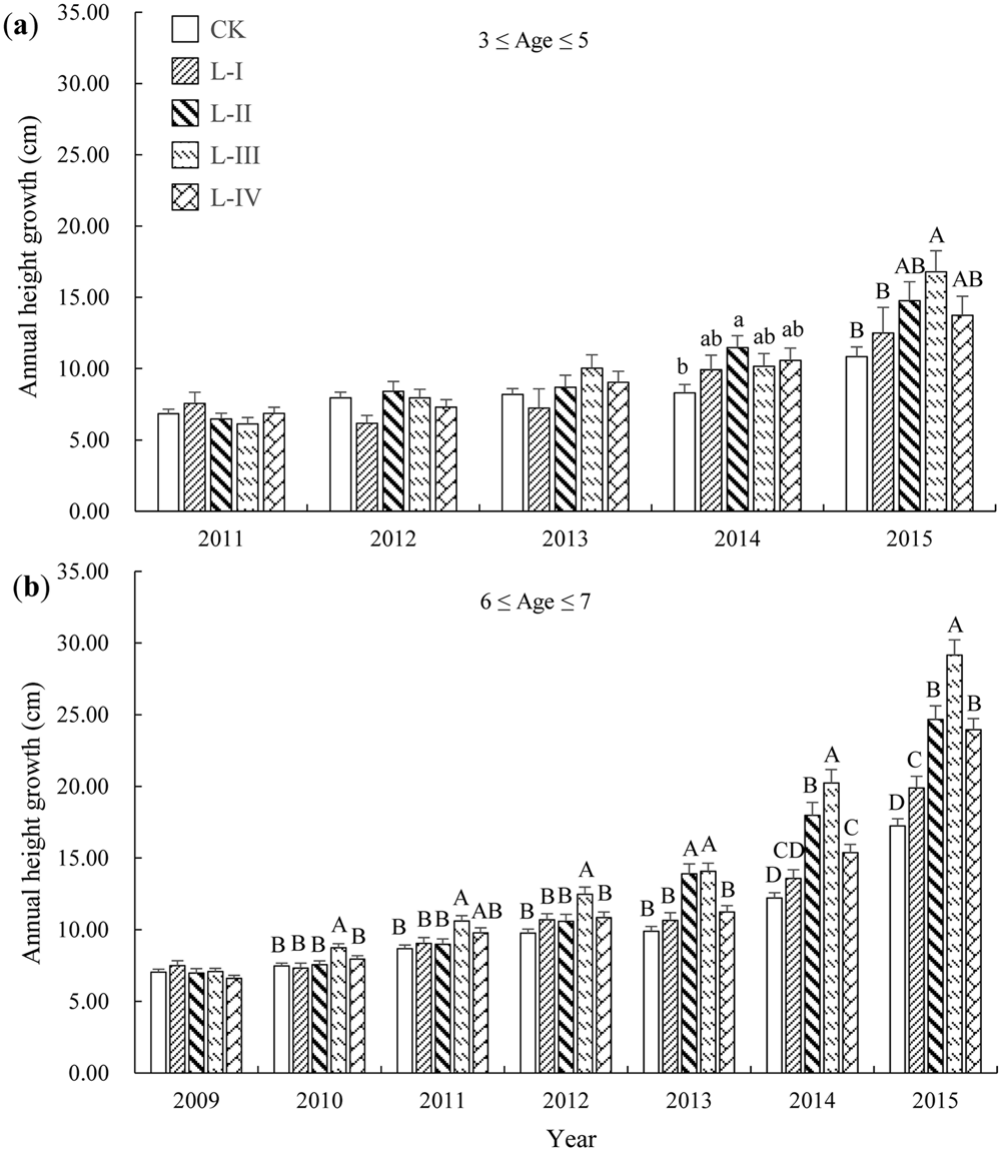
The response of the annual height growth of REAG to gap size. (**a**) REAG of 3 ≤ age ≤ 5. (**b**) REAG of 6 ≤ age ≤ 7. The clustered column diagrams represent the height growth in the same growing season for REAG between different gap size classes. The bars represent ± SE. The uppercase (*p* < 0.01) or lowercase (*p* < 0.05) letters above the standard error bars indicate significant differences (Duncan’s test) among gap size classes for the height growth in the same growing season. Abbreviations are the same as those provided in Fig. 1.

Comparing the height growth of REAG in the same growing season among gap size classes revealed that a significant difference first occurred in 2014 (i.e., four years after germination) for regeneration of 3 ≤ age ≤ 5 (Fig. 5a). In contrast, for regeneration of 6 ≤ age ≤ 7, it appeared in 2010 (i.e., two years after germination) (Fig. 5b). Furthermore, when significant differences in height growth between gap size classes in the same growing season were observed, height growth was found to increase from CK to L-III and then decrease in L-IV; furthermore, the differences between gap size classes increased over time. Consistent with the analysis of the above growth parameters (including crown, CHG, AAH, and AAGD), the maximum and minimum annual height growth values for REAG were found to occur in L-III and CK, respectively (Fig. 5a,b).

**Figure 6:**
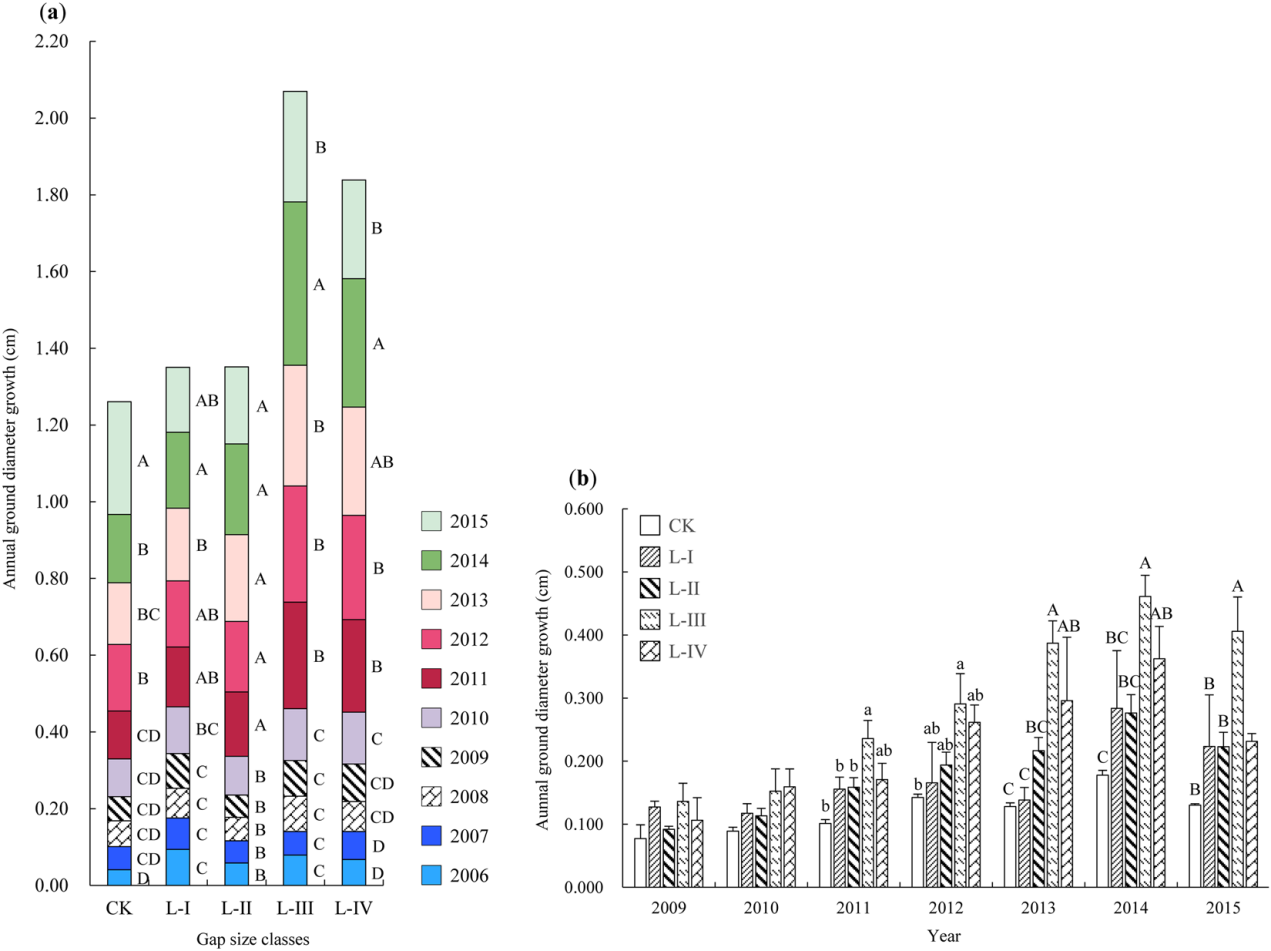
The response of the annual ground diameter growth of REBG (**a**) and REAG (**b**) to gap size. For graphic explanations, refer to Figs 4 and 5.

We also analyzed the annual ground diameter growth and found that the first significant ground diameter growth of REBG occurred in 2012 (i.e., 4 years after gap creation) in CK, whereas it occurred early in 2011 (i.e., 3 years after gap creation) in the other gap size classes (Fig. 6a). In contrast, for REAG, significant differences in the annual ground diameter growth in the same growing season between different gap size classes first occurred in 2011 (i.e., three years after germination). Similar to the annual height growth, these significant differences also increased from CK to L-III and then decreased in L-IV, and the maximum and minimum values were found in L-III and CK, respectively (Fig. 6b).

## Discussion

### Response of age distribution to gap size

The age distribution analysis highlights the present and the past dynamics of regeneration^17^. In this study, the distributions showed an approximately normal distribution across all gap size classes (including CK); these distributions were characterized by a peak at age 7 and the numbers of REAG and REBG decreased when the age was younger or older than this value (Fig. 1). This implies that regeneration in those gaps was unstable.

Based on the age distributions in various gap size classes, we analyzed several factors that may affect the age distribution of Chinese pine regeneration in gaps, as follows: (i) The different ages of parent trees may lead to some differences in the capacity for seed production, which could further affect the germination of seeds, eventually resulting in different age distributions. Wang *et al*.^13^ reported that the age of maximum seed production is typically 30–60 years for Chinese pine, although many unfilled seeds and low germination rates are observed in early periods. Hence, the variation in the number of younger REBG may be the result of increased seed production, from stands with relatively mature ages when the cone yield begins to increase, to the time of gap creation. (ii) The decrease in the number of older REBG over time may be related to shade tolerance. Although they can germinate and grow under shaded conditions during the first three years of their life, Chinese pine is a shade-intolerant tree species. Thus, after the initial growth period, they require more light^18^. Previous studies suggest that an inadequate supply of light may lead to poor growth or mortality, especially for light demanding tree species^6,7,19,20^. Therefore, older REBG may not receive sufficient light and find it difficult to survive, whereas other, younger REBG may survive because of the light provided by gap creation. Similar results were found for *P. thunbergii* by Zhu *et al*.^21^, who reported that seed germination and seedling growth did not require a high-light environment, whereas the subsequent survival and growth of seedlings was largely dependent on light^20^. (iii) The way in which the majority of regeneration were distributed and reached a peak at the year of gap creation may be explained, at least in part, by silvicultural practices, because thinning may have caused a large number of seeds to fall into gaps. Furthermore, the process of logging and removing harvesting residues was likely to scratch the litter layer and expose areas of mineral soil, facilitating contact between seeds and soil and thereby promoting germination^22,23^. During this process, the space and light provided by gap creation are also essential promotors of germination. (iv) The germination of a large number of seeds, the loss of seed vigor in the seed bank, and the reduced seed sources because of parent tree removal from the gap may explain why the number of REAG decreased in the early years after gap creation (as shown in Fig. 1). In addition, (v) the competition between REBG and REAG for available resources and favorable gap positions may also be a potential confounding factor in the age distribution, likely decreasing the establishment and survival of subsequent seedlings. Previous studies have yielded similar results, showing that understory competition hinders the establishment and growth of seedlings but does not affect saplings^1,10,24,25^.

Finally, we determined the coefficient of skewness (*g*) for each frequency histogram of age to correctly predict the direction of regeneration change. These five gap size classes had different skewness coefficients, although they indicated similar age distributions. CK, L-I, L-II and L-III had *g* > 0, indicating the presence of a greater number of larger regenerated pines compared to small individuals in these gaps. In contrast, L-IV had *g* < 0, suggesting the existence of more small trees than large ones, as well as a slow population increase^17^. The decline in population may be attributed to the direct reduction of the number of younger REAG mentioned above. Moreover, we also found that the age distribution skewed toward older individuals (from ages 14 to 18) along the gap size gradient, which was consistent with the changes in the percentages of REBG among gap sizes. These findings provide important evidence suggesting that larger gap sizes promote a wider age range of regeneration and benefit the persistence of older regenerated pines.

### Response of density and growth to gap size

In contrast to some earlier studies^14,26,27^, the density of Chinese pine regeneration was not affected by the gap size and ranged from 1.04 to 1.36 trees m^−2^ in our study. The cause of this phenomenon can be explained, at least in part, by the different proportions of REAG and REBG in each gap size class (Fig. 2). In general, the germination of seeds and the establishment and survival/death of seedlings in gaps are the cumulative results of the characteristics of the tree species, the effects and interaction among environmental factors, and inter- or intra-specific plant-to-plant interactions^28^. Therefore, the variations in the density of Chinese pine regeneration among gap size classes may be linked to multiple factors, including the gap effect. As expected, gap size influenced the growth parameters of regeneration, although no obvious differences in density were observed among gap size classes. These findings also suggested that the responses of growth parameters to gap size increased from CK to L-III and then decreased in L-IV, with the maximum and minimum responses observed in L-III and CK, respectively. Similar trends were observed for the changes in AAH-B and AAH-A of REBG in our study. The AAH-B was lower and exhibited no differences among gap size classes, whereas the AAH-A was significantly higher than AAH-B and showed a variety of responses to gap size (Fig. 3). Based on the above findings, we can conclude that such responses of regeneration growth may be largely attributed to the space and adequate light provided by gap creation. Previous studies have demonstrated that for the growth and development of natural regeneration, especially light-demanding species, light is one of the most important environmental factors^29–32^ and is available through gaps^2,33^. For example, as observed by Gaudio *et al*.^7^ in Scots pine (*Pinus sylvestris* L.), the height of saplings was just 1.7 m in 0–5% full sunlight but 6.1 m in 35–50% full sunlight; indeed, light can account for 46% and 56% of the variation in the height and height increase, respectively. Similarly, other species, such as *Fagus sylvatica*, provide further evidence that the regeneration growing in gaps has a significantly larger diameter, height, and height increase than regeneration under a closed canopy^10,24^.

Compared with the regeneration in L-III, some slight decreases in the growth parameters of regeneration in L-IV were observed, although most of differences between these two classes were not significant. This phenomenon may be related to the following: (i) the photosynthesis and sensitivity to photoinhibition would vary, respectively, if the regeneration was exposed to strong light for a long time, especially under the synergistic effects of light and stress^34,35^; (ii) the growth of heliophytes (such as Chinese pine) growing in a shade environment would be inhibited by suddenly increased light^36^ and a soil surface temperature above 46 °C^18^; (iii) increased light levels can reduce the release of nutrients from decomposition, mineralization and water fluxes, and the nutrient loss becomes obvious when larger gaps are opened^10,37^; and (iv) herbaceous vegetation generally responds quickly to larger gaps, reducing the availability of nutrients and water and thereby inhibiting the growth of woody regeneration^9,24^.

### Response of annual growth to gap size

Gaps influenced age distributions and several regeneration parameters. Regarding whether some degree of response to gap size in the annual height and ground diameter growth of REBG and REAG will occur, our findings indicated that the response of the growth of REBG to gap creation did not occur immediately but exhibited a buffer period known as a response delay (i.e., a buffering effect). The response delay was generally 2–4 years; thus, the annual height increment of REBG began to significantly increase 2–4 years after the gaps were created. For the regeneration established within 1–2 years before gap formation (i.e., regeneration between 8 ≤ age ≤ 9, which accounted for 19.29–41.56% of all regeneration in different gaps), the response delay of growth in CK (3 years) was longer than that in gaps (2 years). In addition, the response delay seemed to increase as the age of REBG increased in our investigated regeneration. For example, regeneration of age ≥ 10 grew slowly and was always suppressed in CK, whereas their growth was delayed by 3–4 years in gaps (Fig. 4). Similar to the annual height growth, a response delay was also found in the responses of annual ground diameter growth to gap creation, with a longer response delay observed in CK (3 years) than in gaps (2 years in all gaps) (Fig. 6a), suggesting that gap creation could promote the growth of regeneration.

Briefly, the height and ground diameter growth of REBG would benefit from the creation of gaps, despite generally delaying the growth therein by 2 to 4 years. A response delay was found not only in gaps but also under the canopy and was prolonged for older regeneration and for regeneration growing under the canopy. These results may be related to the growth pattern of the tree species or the response to the changed environment after gap creation. In a similar study on the growth of Chinese pine, Xu^12^ suggested that the apical growth pattern represents fixed growth and depends on the photosynthetic products produced in previous years; thus, regeneration under the canopy does not respond immediately to gap effects, exhibiting a delay of at least one year after the gaps are opened. This phenomenon is in agreement with findings in *F. sylvatica*, *Acer pseudoplatanus* and *Abies alba*, as confirmed by Vilhar *et al*.^10^. Conversely, in other studies^24,38^ of *F. sylvatica*, the height increase in the regeneration responded directly to opened gaps. However, rapid height growth can occur a long time after gaps are opened, and the response delay depends substantially on the current sizes^7^ and survival conditions of gaps^39,40^, which can readily induce the growth of cones on leader branches to form apical buds and enter eco-dormancy.

Similar to REBG, REAG did not respond immediately to the effects of gaps. In our study, for regeneration established within 1–2 years after gap creation (i.e., regeneration of REAG of 6 ≤ age ≤ 7, which accounted for 39.24–63.99% of regeneration in different gaps), significant differences first occurred in the second year after germination when comparing the height growth in the same growing season among gap size classes. However, the first significant difference was delayed to the fourth year after germination for REAG of 3 ≤ age ≤ 5 (which accounted for 7.17–16.07% of regeneration) (Fig. 5). As we expected, the response delay also occurred in the growth of ground diameter (Fig. 6b) and was generally delayed to the third year after germination. The response delay observed in the growth of REAG among gap size classes could be attributed to (i) the growth characteristics of Chinese pine may be a confounding factor. Xu and Zeng^18^ found that the early stages of Chinese pine primarily focused on underground growth to promote the establishment and survival of regeneration, resulting in slow height growth. (ii) inter- or intra-specific competition cannot be ignored. Because REAG is generally smaller, it is less competitive for light and available nutrients^13,41^.

## Conclusions

We quantified the effects of gap sizes on the age distribution and growth of Chinese pine regenerated before (REBG) and after (REAG) gap creation in a Chinese pine plantation in northern China. Seven years after the gaps were created, our findings showed that the age distribution of regeneration approximated a normal distribution, with the amounts of REAG and REBG decreasing and increasing as the age decreased for all gap size classes, respectively. This pattern of age distribution suggests that regeneration under a canopy or in gaps would exhibit a population decline or regeneration failure. In addition, we found that the gap size exerted no obvious influence on the density of regeneration but significantly affected growth, with maximum and minimum values found in L-III and CK, respectively. Although the response of regeneration growth to gap creation was not immediate, presenting a response delay of 2–4 years, it was ultimately positive. Based on the above findings, which indicate that regeneration can respond vigorously to gap formation, forest managers should consider using gaps to promote canopy regeneration. Subsequent monitoring and gap expansion may be required.

## Material and Methods

### Study site

This study was conducted in the state-owned forest farm in Pingquan County, Hebei Province, China (40°12′N, 115°54′E), characterized by a temperate continental arid monsoon climate with a mean annual temperature of 6.6°C and annual precipitation of 540 mm, of which 60–70% is concentrated in the rainy summer. The soil is mainly composed of 65% forest brown earth and 30% drab soil; and the soil pH value is 6.5–7.5. The main tree species are *P. tabulaeformis* Carr., *Robinia pseudoacacia* L. and *Populus davidiana* Dode., of which Chinese pine plantations compose 8901.85 ha^2^, corresponding to approximately 61.6% of the total forest area of this forest farm. Previously, the natural Chinese pine was the original dominant vegetation in the study site but was seriously deteriorated to grasslands by excessive harvesting. Therefore, the government reforested the area in 1969–1970 at an initial stocking density of approximately 4,950 stems ha^−2^ to reestablish the native vegetation. The stands were managed by a sanitation cutting and a release thinning in 1985 and 1995, respectively. The density was 1,200–1,300 stems ha^−2^; and the average canopy height, diameter at breast height, crown breadth and height of living branches were about 10 m, 13.86 cm, 3.40 m and 2.62 m in 2008, respectively.

### Experimental design

In the winter of 2008, thinning of the study site was conducted by the forest farm using felling machinery and chainsaws. The felled trees primarily included dead and suppressed trees. Logs and slash were removed from the stand. In the spring of 2009, according to the numbers and the size of the gaps surveyed using the line-transect method by randomly setting three lines of 300 meters^16,42,43^, gaps were divided into four size classes: L-I (5–20 m^2^), L-II (20–40 m^2^), L-III (40–60 m^2^), and L-IV (60–100 m^2^); three representative oval-shaped gaps were selected for each size class, respectively. Three square plots of 10 × 10 m were stochastically set up under the canopy as control plots (CK). Four criteria were used for gap selection: (i) a certain amount of Chinese pine regeneration should be growing in the gap; (ii) the gap should be situated at least 50 m from the forest border to eliminate edge effects; (iii) the gap should have been created by the recent thinning (in 2008), and (iv) the distance between any two gaps had to be at least 50 m. The tested gaps in this study were the canopy gaps, and their sizes were estimated as an elliptical area^43^. The mean ( ± SE) sizes of L-I, L-II, L-III, and L-IV were 14.1 ± 2.2, 32.2 ± 2.5, 52.1 ± 3.5, and 86.0 ± 12.8 m^2^, respectively. The Global Positioning System (GPS) coordinates were recorded at the center of each gap to facilitate a follow-up survey (see Supplementary Table S1).

### Data collection

In the summer of 2015, REAG (age ≤ 7) and REBG (age > 7) were investigated in each gap using 5 × 5 m contiguous quadrats^20,44^. In each quadrat, the age, height, ground diameter, crown, and annual height growth of all Chinese pine regeneration were measured and recorded in detail. The age was estimated by counting the whorls or bud scars^13^, and the annual height growth was quantified by measuring the length between two adjacent whorls. The ground diameter was the diameter near the ground surface, which was determined using an electronic vernier caliper. To test the response of the annual ground diameter growth, each of 3 standard regenerated pines were located and marked for REAG and REBG in each gap, respectively. These pines were (i) approximately the average height and ground diameter of each regeneration category, (ii) single stemmed and free of significant disease or herbivore damage, and (iii) not overtopped by nearby regeneration or herbs^45,46^. A cross-section of the stem tissue above the apparent ground diameter was collected from each of the 90 regenerated pines and then dried and polished with sand paper for ring analysis^45,47^. Two perpendicular measurements of annual ground diameter growth were collected with the aid of a microscope. Chinese pine regeneration in gaps and under canopies accounted for 75.9–99.5% of total natural regeneration; therefore, our study focused only on this type of regeneration.

### Data analyses

#### Age distribution

Three repetitions in each gap size class were first combined into one experimental unit (total units, n = 5). To detect the significance, the age distributions in different gap size classes were compared two by two using the Kolmogorov-Smirnov test in SPSS version 17.0. A Monte Carlo simulation with a significance level of 99% was used for precision detection. The coefficient of skewness (*g*), which can correctly predict the direction of population change^48^, was calculated for each age histogram:

1 $$g=\frac{\frac{n\sum {(x-\bar{x})}^{3}}{(n-1)(n-2)}}{{(\frac{\sum {(x-\bar{x})}^{2}}{n-1})}^{3/2}}$$

where *n* is the number of regenerated pines, $x$ is the age, and $\bar{x}$ is the average age.

*g* > 0 indicates a distribution with few younger regenerat ion and many older regeneration, whereas *g* < 0 indicates a distribution with few older regeneration and many younger ones^17^. A logarithmic transformation was performed for age before calculating *g*. In addition, to understand the changes between these two age groups of regeneration, the percentages of REAG and REBG were compared in the same gap size class and in different gap size classes. Analysis of variance (ANOVA) and multiple comparisons (Duncan’s test) were implemented using SPSS version 17.0. Significant differences were detected at *p* < 0.05.

#### Differences in density and growth

Chinese pine regeneration was determined by height, ground diameter, crown, current height growth (CHG), annual average height growth (AAH) and ground diameter growth (AAGD). The density and mean growth parameters were calculated in each gap and then analyzed by ANOVA. These statistical analyses were conducted in SPSS version 17.0 using a single-factor design, and the means of significant treatment effects were separated using Duncan’s multiple range test. All statistical tests were conducted at an alpha level of 0.05. The differences in density and growth for ALL (all regeneration in a gap), REAG and REBG in different gap size classes were analyzed by repeating the procedure described above. We also compared the AAH-B (the annual average height growth before gap creation) and AAH-A (the annual average height growth after gap creation) for REBG in the same gap size class and in different gap size classes.

#### Responses of annual height and ground diameter growth

To gain insight into past and current growth trends of regeneration, both annual height and ground diameter were analyzed in each gap. Furthermore, to minimize the effects of age on annual growth, the age classes were assigned according to the frequency histograms of age displayed in Fig. 1: REAG (*j*_1_ ≤ age ≤ 5 and 6 ≤ age ≤ 7) and REBG (8 ≤ age ≤ 9, 10 ≤ age ≤ 12, and 13 ≤ age ≤  *j*_2_), where *j*_1_ and *j*_2_ were the minimum and maximum ages of selected samples, respectively. Since the amounts of regeneration with age < 4 years or >14 years were scarce (Fig. 1), the selected numbers of the defined minimum (*j*_1_) and maximum ages (*j*_2_) were more than three specimens. All regeneration in each gap size class was first merged to form an experimental unit (n = 5), and then, the height growth of each growing season in each age class was averaged. For these 5 size-classes gaps, the growth of regeneration was thus separately calculated and analyzed. Similarly, the analysis procedure for annual ground diameter growth was the same as that of annual height growth. The difference was that the annual ground diameter growth was based on 45 samples (3 specimens × 5 gaps × 3 repetitions) for REAG and REBG each, whereas the measurement of annual height growth included all regeneration in each gap size class.

As defined above, REBG describes the regeneration which have survived for one or more years under a dense canopy prior to the creation of gaps and, as a result, experienced a change in their micro-environment caused by gap creation. In contrast, REAG describes regeneration that was established after gap formation. Therefore, in our study, the differences in the annual height or ground diameter growth of REBG are displayed using stacked graphs, and the results obtained for the same gap size class are compared between growing seasons. In contrast, the differences in the annual height or ground diameter growth of REAG are displayed as column diagrams, and the results found in the same growing season are compared between gap size classes. For REBG, if the height or ground diameter growth at the beginning of gap creation (for example, 1 year after the gap creation) was immediately significantly greater than that of every year before gap creation, the height or ground diameter increment was considered to have directly responded to gap creation; otherwise, it was described as exhibiting a response delay. The response delay was equal to the difference between the year in which the first significant height or ground diameter growth occurred and the year of gap creation. For the growth of REAG among gap size classes, if the first significant difference in the height or ground diameter was observed in the same growing season, then the difference between the year corresponding to the first significant difference and that of germination was regarded as the age at which REAG began to show significant differences. ANOVA and Duncan’s test (alpha = 0.05) were implemented in SPSS version 17.0.

## Electronic supplementary material

Table S1
