# Supplementary material for: Various response of Pinus tabulaeformis Carr. regeneration in artifical gaps
Source: Sci Rep. 2017 Nov 6;7:14568. doi: 10.1038/s41598-017-15322-8 (PMC5673939; doi:10.1038/s41598-017-15322-8)
Supplement: Supplementary file 1 — Table S1 [file 41598_2017_15322_MOESM1_ESM.pdf]

Supplementary Information

Various response of *Pinus tabulaeformis* Carr. regeneration in artifical gaps

Zhibin Wang, Kuangji Zhao, Haijiao Yang, Lvyi Ma, and Zhongkui Jia

Table S1. GPS coordinates and elevation of each studied gap (taken at gap center). See main text for abbreviations.

Gap size	Repetition 1		Repetition 2		Repetition 3	
classes	Elevation (m)	GPS	Elevation (m)	GPS	Elevation (m)	GPS
CK	723	41°15'259"N	707	41°15'243"N	716	41°15'380"N
		118°46'808"E		118°46'835"E		118°46'814"E
L-I	706	41°15'280"N	706	41°15'255"N	703	41°15'261"N
		118°46'865"E		118°46'843"E		118°46'847"E
L-II	712	41°15'296"N	718	41°15'296"N	711	41°15'286"N
		118°46'815"E		118°46'802"E		118°46'826"E
L-III	711	41°15'268"N	704	41°15'271"N	717	41°15'302"N
		118°46'830"E		118°46'843"E		118°46'808"E
L-IV	695	41°15'272"N	714	41°15'276"N	723	41°15'304"N
		118°46'849"E		118°46'861"E		118°46'797"E
